# Farmer Health and Adaptive Capacity in the Face of Climate Change and Variability. Part 1: Health as a Contributor to Adaptive Capacity and as an Outcome from Pressures Coping with Climate Related Adversities

**DOI:** 10.3390/ijerph8104039

**Published:** 2011-10-21

**Authors:** Helen L. Berry, Anthony Hogan, Suan Peng Ng, Anne Parkinson

**Affiliations:** 1 Centre for Research and Action in Public Health, The University of Canberra, University Drive, Bruce, ACT 2601, Australia; E-Mail: helen.berry@canberra.edu.au; 2 School of Sociology, College of Arts and Social Sciences, The Australian National University, ACT 0200, Canberra, Australia; E-Mail: anneparkinson@australiaonline.net.au; 3 Adaptation Research Network for Human Health, and National Centre for Epidemiology and Population Health, The Australian University, ACT 0200, Canberra, Australia; E-Mail: suanpeng.ng@anu.edu.au

**Keywords:** climate change, farmer health, adaptive capacity

## Abstract

This paper examines the role farmers’ health plays as an element of adaptive capacity. The study examines which of twenty aspects of adaptation may be related to overall health outcomes, controlling for demographic and on-farm-factors in health problems. The analysis is based on 3,993 farmers’ responses to a national survey of climate risk and adaptation. Hierarchical linear regression modelling was used examine the extent to which, in a multivariate analysis, the use of adaptive practices was predictively associated with self-assessed health, taking into account the farmer’s rating of whether their health was a barrier to undertaking farm work. We present two models, one excluding pre-existing health (model 1) and one including pre-existing health (model 2). The first model accounted for 21% of the variance. In this model better health was most strongly predicted by an absence of on-farm risk, greater financial viability, greater debt pressures, younger age and a desire to continue farming. Social capital (trust and reciprocity) was moderately associated with health as was the intention to adopt more sustainable practices. The second model (including the farmers’ health as a barrier to undertaking farm work) accounted for 43% of the variance. Better health outcomes were most strongly explained, in order of magnitude, by the absence of pre-existing health problems, greater access to social support, greater financial viability, greater debt pressures, a desire to continue farming and the condition of on-farm resources. Model 2 was a more parsimonious model (only nine predictors, compared with 15 in model 1), and explained twice as much variance in health outcomes. These results suggest that (i) pre-existing health problems are a very important factor to consider when designing adaptation programs and policies and (ii) these problems may mediate or modify the relationship between adaptation and health.

## 1. Introduction

The most recent research on farmer health reports that this cohort is “at higher risk of physical and mental illness when compared to rural and metropolitan populations” [1]. Brumby *et al.* report that farmers:

“face an environment of high occupational hazards, poor access to health services, higher mental health burden, vulnerability to adverse climatic conditions, socio-economic constraints, food insecurity, alcohol misuse and an increasing burden of chronic disease” [1].

The specific health risks faced by farmers have significantly intensified over the past 10 years as Australia has experienced drought, increased drying [2] and greater compressing terms of trade in agriculture [3]. Berry *et al*. [4] report that “Australia has the world’s most variable climate” and that “(R)ural vulnerability to mental health problems is greatly increased by socio-economic disadvantage”; disadvantage which results from climatic and economic impacts. Berry *et al*. [4] also observe that while “mental health problems might not be elevated among farmers as a group, relative to the general population (...) averaged survey scores may mask systematic variation between types of farmer”. Climate variability contributes to socio-economic vulnerability through its impacts on productive capacity and therefore income. Stressors associated with the unpredictability of climate and income in turn contribute to mental health vulnerability [5]. Vulnerability to the impacts of adverse climate change arises when risks are high and the capacity to adapt is low [6]. The Intergovernmental Panel on Climate Change (IPCC) [7] defined general adaptive capacity in terms of “income, education and health” noting that “human and social capital are key determinants of adaptive capacity at all scales, and that they are as important as levels of income and technological capacity” [7]. Central to the idea of adaptive capacity is the ability to cope with and adapt to adverse changing environmental conditions [8–12]. Key determinants include the ability to learn, store knowledge and experience, and approach problem solving in creative, flexible and novel ways [13–15]. Also beneficial is the ability to experiment with new and novel solutions, and take on board a wide range of challenges [16].

Health is an essential component of the capacity to adapt to climate change and psychological health is an essential component of resilience [17]. Resilience is that reservoir of personal psychological coping assets and social capital that provide people with the will and the mental toughness to make necessary changes in the face of severe and continuing adversity. Climatic and subsequent economic events appear to undermine resilience by eroding opportunities for social processes which maintain social capital and support:

“when family farms are struggling with events such as dryness, the communities in which people normally spend their money and participate also suffer. Dryness negatively impacts on the ability of members of a rural community to work together for the benefit of the whole community, eroding the capacity of people to engage in community projects or do the voluntary work that keeps rural communities alive” [17].

Coupled with resilience is the farmers’ sense of identity and life purpose. A person’s sense of their personal and social identity provides them with meaning and life orientation. These factors orient their behaviors to specific ends which provide them with the will and determination not just to make necessary changes, but which in fact serve to underpin the decision to persist with current behaviors despite their consequences. This approach to identity has been associated with on farm adaptive decision making [18,19].

Daffara *et al*. [20] report that while quite a lot of work has gone into describing what adaptive capacity might be, less research has focused on the relationship between adaptive capacity and the adoption of adaptive practices. Notably the role of health in relation to adaptive capacity is not well researched. This study is presented in two parts. Part 1 of this study is concerned with the extent to which farmers’ health may be predictively associated with adaptive decision making, as well as an outcome of, climate related circumstances. Part 2 of this paper extends this work and considers potential differences in profiles of farmers, when farmer health is considered within the context of the factors which influence how farmers may adapt.

## 2. Methods

*Data*. The data used in this study were taken from a larger study of Australian farmers’ adaptation to the challenges of climate change conducted by the Department of Agriculture, Fisheries and Forestry (DAFF) in Australia in 2008 [21,22]. It was a representative cross-sectional sample of 3,993 Australian farmers who voluntarily completed a mailed questionnaire. The representativeness of the sample was validated against the Australian Bureau of Statistics Farm Survey (see [21] and [22] for further details). The survey contained 115 items based on nine themes (condition of farm, on-farm adaptation, interest in alternative energy forms, attitudes to climate change, perceptions of climate impact, aspects of social capital, program participation, information usage and aspirations for future programs). The survey was designed to capture information concerning each of these themes, or concepts, derived as they were from antecedent qualitative studies [2] which also took into account the various multi-factorial approaches to adaptation noted in the literature [24–28], natural resource management and farmer typologies [19,29–31]. These themes were supplemented with 11 demographic items such as farm size, type of agricultural production, irrigation status, age, gender, and on- and off-farm income. At the time the data were collected, many parts of Australia were experiencing their worst drought on record.

Following the approach taken in the antecedent qualitative research [23], we operationalized adaptation to climate change in two ways. The first concerned the *utilization of planned risk management* strategies put in place with the intention of responding to the *shorter and longer-term impacts of climate change*. These strategies encompassed activities such as: diversification of production and income-earning; taking up training; implementing a variety of risk management programs (e.g., with respect to business, hazards and operational considerations); and downsizing strategies such as selling or leasing property or leaving the industry. The second concerned the *intention to adapt*. This intention encompassed interest in using the property to earn carbon credits, to reduce emissions and to adopt more sustainable practices.

### 2.1. Twenty Key Concepts

The questionnaire designed to collect the data for this study contained a large number of items intended to capture information around a number of themes as described above, [23]. In recognition that these concepts were likely to be multifaceted, the questionnaire contained multiple items (questions) for many of these concepts. Our first task was to examine whether the data provided empirical support for these concepts and, if so, to develop and refine composite measures for each of them.

We thus began with analyses to examine the concepts contained in the dataset. Our intentions in doing so were:

- to reduce the N = 126 items in the dataset to a manageable number of composite variables that would be dense (like the concepts they were designed to measure), informative and meaningful (this also reduces errors in the later analyses);
- to test the concepts to ensure that we were measuring what we intended to measure;
- to create accurate, weighted composite variables for each concept that included *all* and *only* statistically valid items; and
- to produce concise, accurate definitions of these concepts measured in a way that would reflect their multi-faceted nature.

To do this, we adopted a three-phase approach. First, we undertook exploratory factor analyses (see Box 1) of groups of items that had been included in the survey to tap the nine separate themes identified in the original survey instrument. Exploratory factor analyses helped identify the structure of complex underlying or “latent” concepts, thereby indicating how many concepts the dataset contained, which items “belonged” in the concept, and to what extent they were representative of that concept (that is, how heavily they load statistically on the factor). Our exploratory factor analyses suggested the presence of twenty underlying (latent) concepts.

Box 1 **Exploratory factor analysis: background information and particulars of the present study**

The purpose of exploratory factor analysis is to explore the underlying structure of a large quantity of data where these data are intuitively related. The data for the present study met the conditions for exploratory factor analysis: the study was originally designed in such a way as to be suitable for exploratory factor analysis (multiple items tapping each concept); the variables were intuitively related; the dataset was factorable (the majority of correlations were >0.30); the sample size was “excellent” (more than 10 respondents per item to be factor analyzed in the dataset). The principal criteria for evaluating the factor solutions were (i) meaningfulness and interpretability (factors that made sense and were consistent with the literature), (ii) scientific usefulness, (iii) parsimony, and (iv) fewer than 5% non-redundant residuals. Exploratory factor analysis was performed on the data to examine the factor structure underlying the items. The sampling statistics: Kaiser-Meyer-Olkin statistics (KMO = 0.921) and Barlett’s test of sphericity (p < 0.001) indicated that the dataset was appropriate for factor analysis. This was further indicated by the adequate sample size (n = 3,993, or >the number of variables to be factor analyzed times ten) and factorable data (a large proportion of correlations >0.30). Maximum likelihood factoring with oblimin rotation were used in the analysis as they are designed, respectively, to allow for non-normally distributed data and correlated factors.

The next step was to test, or confirm, the validity of these twenty concepts and the reliability of individual items loading on them. We did this by conducting twenty one-factor congeneric modelling analyses, that is, one for each latent concept. These models test, remove error from and refine the underlying structures (latent concepts) suggested by the exploratory factor analyses.

Of particular note, the one-factor congeneric modelling analyses permit the accurate definition and naming of each concept, so that each concept may be clearly understood. In our third and final step, we used weightings derived from the one-factor congeneric models to create valid and accurately weighted composite scores for each concept in our study.

We were able to confirm the presence of the twenty latent concepts that we identified in step one and to refine their structure and measurement. This gave us a set of twenty accurately named and measured concepts on which to perform the substantive analyses for this study. These concepts are summarized in Table 1. Further information about these analytic techniques may be found in Berry and colleagues [32,33]. We included in our analyses all twenty of the weighted latent constructs that we derived from exploratory factor analysis and one-factor congeneric modelling.

### 2.2. Other Measures

The Kenny Report [17] concluded that, in the context of the multiple pressures farmers were facing, drought was “the last straw” for farmers. Consistent with this concept, we computed a mean index that was based on farmers’ responses to 16 items listing the pressures they were facing and which were potentially problems in managing their properties. These pressures are listed in Table 1 (items which contribute to concepts one through four) and take into account a variety of factors such as input costs and commodity prices, debt levels, interest rates, cash flow, farm income, labor, access to services and training, resource conditions and personal health. Note that “my health and fitness” was one item on this list. We did not include this item in the index because we analysed it separately as a health-related item.

### 2.3. Five Overarching Concepts

The twenty weighted composite measures for the latent concepts identified and refined through the exploratory factor analysis and one-factor congeneric modelling were then subjected to a second-order exploratory factor analysis (Box 2). That is, a second exploratory factor analysis was performed on the twenty composites developed from the initial factor analysis and then subjected to one-factor congeneric modelling.

Box 2 **Second-order exploratory factor analysis**

Inspection of the correlation matrix revealed many coefficients of 0.30 or above. The Kaiser-Meyer-Oklin value was 0.765, exceeding the minimum recommended value of .6 (Kaiser, 1970, 1974) and Bartlett’s test of sphericity (Bartlett, 1954) reached statistical significance (*p* < 0.001) indicating that the twenty latent constructs were appropriate for exploratory factor analysis. The results revealed the presence of five factors with eigenvalues exceeding 1, which explained 53.33 percent of the total variance. The sixth factor had an eigenvalue of 0.938. Examination of the screeplot identified a clear break after the fifth factor.

The purpose of this was to reduce (summarize) the twenty concepts to a more manageable number for use in the cluster analysis which is reported in Part 2 of this study [34]. This second-order analysis produced more accurate, meaningful and interpretable results with fewer (higher order) input variables. As there was considerable overlap (bivariate correlation) between the twenty concepts, it was appropriate to summarize these in this way for this purpose. This second order factor analysis produced the following five factors which can be interpreted as overarching concepts:

- Belief in climate change.
- Desire for financial assistance and advice.
- Social connectedness.
- Information-seeking.
- Adverse farm conditions.

These factors were saved as weighted standardized composite scores and used in the cluster analysis. The respective loadings for each of the factors can be seen in Table 2.

We approached health as a precipitating factor, *i.e.*, one that could contribute to decision-making (for example, poor health could make it more likely that a farmer might withdraw from farming) *and* as an outcome resulting from the pressures associated with coping with climate-related adversities. That is, we hypothesized that health is equally a *contributor to* and *outcome of* complex agricultural adaption systems. We therefore included items tapping health in two different analyses. There were two such items: the *predictor* item “my health and fitness” (as a barrier to running the property), which was one of the items included in the list of pressures farmers may be facing; and the *outcome* item “my health is good”. Some further explanation of this approach is warranted. We were not primarily interested in how past health affects present health (except to control for it statistically) as this is already known. That is, put simply, one’s past health is a good guide to one’s present health. Instead, we wanted to produce new knowledge about: (i) which aspects of adaptation are related to health outcomes; (ii) taking account of relevant aspects of adaptation, how existing health problems may affect health outcomes; and (iii) whether including existing health problems in the analysis may modify which aspects of adaptation are related to health outcomes and, if so, (a) the magnitude of the relationships and (b) possible implications for program and policy development (which are considered in the discussion section of this paper).

### 2.4. Other Measures Used in the Study

In addition to creating weighted composite scores and second-order factors for use in our analyses, we also included data on *socio-demographic characteristics* (sex, years of education post-Year 10, on-farm and off-farm income, self-assessed financial viability and age) and *farming-related variables* (land size, irrigation status and main area of primary production).

### 2.5. Hierarchical Linear Regression Models

**Figure 1:**
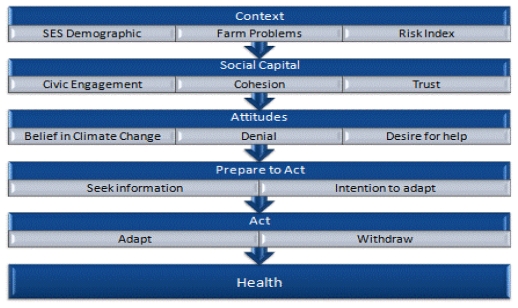
Hierarchical approach to modelling planned approaches to predicting farmer health.

This paper reports two hierarchical models which we conducted. In each of these models, we entered variables in blocks according to our hypotheses (Figure 1, and described below) using the health variable (*“my health is good”*) as the dependent variable. This *health impacts* model was considered in two ways, with and without the inclusion of the predictor variable *“my health and fitness”* (as a barrier to operating the property). This was undertaken to test two distinct hypotheses; the first examined the extent to which, in a multivariate analysis, the intention to adapt together with planned approaches to risk management were predictively associated with self-assessed health. The second analysis examined the extent to which the intention to adapt and planned approaches to risk management were predictively associated with self-assessed health, *taking into account* the farmer’s rating of whether their health was a barrier to undertaking farm work.

## 3. Results

The tables below present unstandardized BETA and standardized *beta* values derived from each of the hierarchical regression analyses showing all (and only) the variables that made a significant independent contribution to explaining variance in the outcome variable for each model. As noted above, each regression analysis controlled for socio-demographic factors (sex, age, education and income). Included in the models reported here (and in this order) were factors concerned with: social capital; climate change (belief in, evidence of, denial of); desire for government help; adaptive intentions; information-seeking behaviours; and financial viability. The items making up these factors can be found in Table 1. Full details of each of these models, including variables that did not contribute significantly to explaining variance, can be obtained from the authors.

### 3.1. Farmer Health as Predicted by Risk Management and Adaptation Practices

Table 3 provides the final unstandardized BETA and standardized *beta* values derived from the first hierarchical regression analysis showing all (and only) the variables that made a significant independent contribution to explaining variance in farmer self-rated health (*“my health is good”*). We controlled for socio-demographic factors and took account of planned approaches to risk management and the intention to adapt. The model (which is statistically significant at *p* < 0.001) explained 21% of variance in farmer self-rated health. In the final model, better health was most strongly predicted by: an absence of on-farm risk, greater financial viability, greater debt pressures, younger age and a desire to continue farming (rather than to withdraw). Greater sense of belonging, higher levels of reciprocity and trust, taking up adaptive practices and the absence of feelings of moral responsibility for climate change also made very small but nonetheless significant independent contributions to explaining variance in (better) farmer health.

Table 4 provides the final unstandardized BETA and standardized *beta* values derived from the second hierarchical regression analysis showing all (and only) the variables that made a significant independent contribution to explaining variance in farmer self-rated health (*“my health is good”*). In this analysis, we re-ran exactly the model described immediately above, but this time added the farmer’s assessment of their health and fitness to work as a predictor variable. The model (also statistically significant at *p* < 0.001) explained 43% of variance in farmer health. Variance in farmer self-rated health was most strongly explained, in order of magnitude, by: health and fitness not being a barrier to farming; greater access to sufficient social support; greater financial viability; greater debt pressures; and a desire to continue farming (rather than to withdraw). Two other social factors also made a small but nevertheless significant contribution to explaining variance in farmer self-rated health: in order of magnitude, sense of belonging and reciprocity.

## 4. Discussion and Conclusions

This study examined human health both as a contributor to practice and as an outcome of climate change-related circumstances. The study drew on data from a substantial national survey of farmers that investigated their health and adaptive capacity. Given the cross-sectional nature of the study design, causal inferences cannot be drawn and it is not our intention to do so. A number of statistical associations were identified in the analysis and these are discussed here. As a contributor to practice, farmers with poorer health were, as we expected, more likely than were their peers to report that their health was a barrier to sustaining work on the farm. This is a significant finding particularly because, as the farming population ages, (old) age itself might be a barrier to remaining in the business and to adapting to climate change [35]. We found this to be the case, *but only for those reporting poorer health*. For older farmers with good health, age did not contribute to their adaptation decisions. Turning to health as an outcome of climate change-related circumstances, in order of the importance of farming business-related factors, farmers who reported fewer debt pressures, were confident in their financial viability, were younger and wished to continue farming reported better health than did other farmers. Further, farmers with greater social support, sense of belonging, trust and reciprocity also reported better health than did their less-connected peers.

Some important implications arise from comparing the results of the two separate analyses. Model 1 (which did not include health problems as a predictor variable) revealed that, taken together, 15 adaptation-related variables could explain 21% of variance in health. Model 2 revealed that, with the addition of health problems as a predictor variable, 9 adaptation-related variables could explain 43% of variance in health and that a different set of predictors explained health outcomes. Of the 15 variables that were retained in Model 1, the following were also retained in Model 2: debt pressures; support with problems; sense of belonging; reciprocity; adaptive practices; financially viable; and withdrawing. The variables of: age, years of education, barriers, market pressures, crude risk index, trust, moral responsibility for climate change and adaptation intentions were not able to be retained in Model 2. Model 2 also varied from Model 1 in that the condition of the natural resource (*resources*) was retained in Model 2 but not in Model 1.

From this, we can conclude that the model including health problems as a predictor variable was both a more parsimonious and, therefore, more pragmatically useful model (using only 9 predictors, compared with 15 in Model 1) and that, also, it explained twice as much (43% *vs.* 21%) variance in health outcomes, rendering it a much more powerful model overall. These findings suggest that (i) existing health problems are a very—perhaps the most—important factor to consider when designing adaptation programs and policies for farmers and that (ii) existing health problems may mediate or modify the relationship between adaptation and health. This latter proposition requires further investigation.

The literature reviewed in this paper proposed that health was an important component of adaptive capacity. The literature showed that farmers already have poorer health than other Australians (perhaps partly to do with their greater average age) and that this health status, particularly mental health, was undermined by socio-economic disadvantage arising in association with climatic impacts. The literature also argued that adaptive capacity was an essential component of people’s ability to take on new challenges and solve problems. At the same time, claims are widely published that resilience and social capital are essential to mental toughness and the determination to make necessary changes. The findings of this study support the view that health is an essential component of adaptive capacity—and that it is also powerfully influenced by climate-related farm pressures.

In this study, health was associated with adaptive capacity evidenced by the ability to remain in business and to adapt to climate change. The financial viability of farms, a factor greatly affected in Australia by climate variability, was associated with farmer health as were psycho-social aspects of wellbeing, such as social support, sense of belonging, trust and reciprocity. This study makes more evident the links that other studies have proposed to underpin these associations. Brumby *et al*. [1] argue that the interplay of climate and economic vulnerability exacerbates existing farmer vulnerability and contributes to a defeat cycle through increased exposure to physical and psychological health risks. From the health perspective, two factors appear critical to enabling farmers to develop more effective adaptation strategies: (i) the need to understand that their health may be profoundly affected [36] by the inter-play of climatic and economic events and that this may, just as profoundly, affect their capacity to cope and adapt, and (ii) the need to support farmers to increase their personal care [1] while maintaining effective social support [4], which in turn is seen to reduce the effects of such stressors.

## Figures and Tables

**Table 1:** Summary of twenty latent factors and the variables that loaded on them.

Factor	Items contributing to factor
1. Barriers to accessing support services	Not enough access to community services Lack of access to training or professional services Cost of training or professional services
2. Debt pressures	Interest rates, cash flow, debt levels
3. Condition of on-farm resources	Water quality, soil quality, pests and diseases
4. Market pressures on farm viability	Low commodity prices Input costs (fuel, energy and fertilizer costs)
5. Adaptation through planning and managing property (risk management)	Diversify into other forms of production Improve financial situation (improve cash flow, restructure debt) Develop risk management strategies for natural hazards Develop a business management plan Undertake training to improve on farm income Use operational management plan (crop rotation, plan stock numbers) Succession planning
6. Intention to withdraw from farming	Sale back operations Sell or lease part of the property Exit the industry
7. Intention to adapt practices	Interest in using property for earning carbon credits Interest in using new technologies to reduce emissions from livestock/fertilizer use Interest in adopting more sustainable land management practices
8. Desire to produce greenpower	Interest in having wind turbines on property for energy production Interest in having hydro power on property for energy production Interest in having solar panels on property for energy production
9. Sense of moral responsibility to act to reduce greenhouse gas emissions	Some farming practices generate greenhouse gas emissions (GHEs) The community has a moral responsibility to reduce GHEs It’s the government’s responsibility to legislate to reduce GHEs I have a responsibility to reduce GHEs
10. Belief in climate change	There is no such thing as climate change GHEs cause climate change Climate patterns are really changing The increased intensity of droughts, storms and floods is a result of climate change
11. Belief in climate change	There is no such thing as climate change GHEs cause climate change Climate patterns are really changing The increased intensity of droughts, storms and floods is a result of climate change
12. Belief in climate change	There is no such thing as climate change GHEs cause climate change Climate patterns are really changing The increased intensity of droughts, storms and floods is a result of climate change
13. Financial viability of the property	Not enough farm income to support the family Changes in weather patterns are hurting my business Climate change is threatening the viability of my property
14. Physical evidence of climate change	Local changes in weather (e.g., less rain, more dust storms, warmer temperatures) Shift in seasons (e.g., earlier/later frosts) Reduced availability of water on my property The melting of ice bergs
15. Confidence in coping ability	Thanks to my resourcefulness, I can handle unforseen situations I can remain calm when facing difficulties because I can rely on my coping abilities If I am in trouble, I can think of a good solution I can cope with more change
16. Trust	Most people can be counted on to do what they say they will Most independent experts can be relied upon to tell the truth about the limits of their knowledge I can trust people in government to look after my interests
17. Receiving direct government financial support	Exceptional Circumstance Interest Rate Subsidy Exceptional Circumstance (EC) Relief payment Professional advice and planning through EC Rural financial counselling program
18. Advice from rural organizations	Agricultural extension programs or advisors Landcare/Caring for Country programs Non government groups Regional natural resource or catchment management groups
19. Desire for government initiatives to promote adaption to sustainable farming	Enable me to develop more sustainable practices Enable me to access advice and support for farm and natural resource management Provide me with information on water allocation and availability
20. Desire for direct government financial assistance	Provide me with direct financial assistance to manage current problems Provide me with direct financial assistance to enable me to invest in the property’s long-term future
21. Access to on-line information (sources used)	Bureau of Meteorology Weather forecasting services Internet (e.g., Google)
22. Access to information via non-on-line sources (sources used)	Media (TV, Radio, Print) Industry associations and groups Farm journals and rural press Word of mouth

**Table 2:** Results of the second-order factor analysis: Five overarching factors.

Concept	Belief in climate change	Desire for financial assistance and advice	Social Connectedness	Information seeking	Adverse farm conditions
Notice evidence of climate change	0.872				
Believe climate change is real	0.871				
Moral responsibility to reduce GHEs	0.715				
Concern about financial viability in the face of climate change	0.583				
Financial help and advice		−0.804			
Offering direct financial assistance		0.685			
Debt pressures		0.523			
People help each other out (reciprocity)			0.829		
I feel part of my local community			0.790		
I have people to assist with problems			0.653		
Confidence about coping			0.429		
Trust			0.366		
Non-electronic information sources about weather/climate				−0.754	
Risk Management (actively managing multiple pressures)				−0.981	
Seeking advice from rural organizations				−0.669	
Help make my farming practices more sustainable				0.565	
Condition of on-farm resources					0.749
Barriers to accessing support services					0.745
Market pressures on farm viability					0.440

**Table 3:** Predictors of farmer self reported health.

Final model	B	Std Err B	*β*	*R^2^*
Age	−0.01	0.00	−0.11 ***	0.21 ***
Years of Education after Year 10	0.02	0.01	0.04 *	
Barriers	0.04	0.02	0.04*	
Debt pressures	0.17	0.02	0.18 ***	
Market pressures	0.08	0.03	0.05 **	
Crude risk index	−0.40	0.04	−0.26 ***	
Support with problems	0.22	0.02	0.19 ***	
Sense of belonging	0.06	0.02	0.06 **	
Reciprocity	0.05	0.02	0.05 **	
Trust	0.04	0.02	0.03 *	
Moral responsibilities	−0.04	0.02	−0.04 *	
Adaptive practices	0.06	0.02	0.05 **	
Financially viable	0.19	0.01	0.20 ***	
Adapt	0.04	0.02	0.04 *	
Withdrawing	−0.12	0.02	−0.11 ***	

* p < 0.05;

** p < 0.01;

*** p < 0.001.

**Table 4:** Predictors of farmer self reported health controlling for current health and fitness.

Final model	B	Std Err B	*β*	*R*^2^
My health/fitness	0.47	0.01	−0.55 ***	0.43 ***
Debt pressures	0.10	0.01	0.11 ***	
Resources	0.06	0.01	0.06 ***	
Support with problems	0.20	0.02	0.17 ***	
Sense of belonging	0.06	0.02	0.05 **	
Reciprocity	0.06	0.02	0.05 ***	
Adaptive practices	0.05	0.01	0.05 ***	
Financially viable	0.14	0.01	0.15 ***	
Withdraw	−0.04	0.01	−0.04 **	

* p < 0.05;

** p < 0.01;

*** p < 0.001.
